# Bioinformatic pipelines for whole transcriptome sequencing data exploitation in leukemia patients with complex structural variants

**DOI:** 10.7717/peerj.7071

**Published:** 2019-06-12

**Authors:** Jakub Hynst, Karla Plevova, Lenka Radova, Vojtech Bystry, Karol Pal, Sarka Pospisilova

**Affiliations:** 1 Central European Institute of Technology, Masaryk University, Brno, Czech Republic; 2 Department of Internal Medicine—Hematology and Oncology, Faculty of Medicine, Masaryk University, Brno, Czech Republic; 3 Department of Internal Medicine—Hematology and Oncology, University Hospital Brno, Brno, Czech Republic

**Keywords:** Chromothripsis, Complex structural variants, Fusion gene, Gene expression, Bioinformatic pipeline, Next-generation sequencing, Leukemia, Transcriptomics, Chronic lymphocytic leukemia, Statistics

## Abstract

**Background:**

Extensive genome rearrangements, known as chromothripsis, have been recently identified in several cancer types. Chromothripsis leads to complex structural variants (cSVs) causing aberrant gene expression and the formation of *de novo* fusion genes, which can trigger cancer development, or worsen its clinical course. The functional impact of cSVs can be studied at the RNA level using whole transcriptome sequencing (total RNA-Seq). It represents a powerful tool for discovering, profiling, and quantifying changes of gene expression in the overall genomic context. However, bioinformatic analysis of transcriptomic data, especially in cases with cSVs, is a complex and challenging task, and the development of proper bioinformatic tools for transcriptome studies is necessary.

**Methods:**

We designed a bioinformatic workflow for the analysis of total RNA-Seq data consisting of two separate parts (pipelines): The first pipeline incorporates a statistical solution for differential gene expression analysis in a biologically heterogeneous sample set. We utilized results from transcriptomic arrays which were carried out in parallel to increase the precision of the analysis. The second pipeline is used for the identification of *de novo* fusion genes. Special attention was given to the filtering of false positives (FPs), which was achieved through consensus fusion calling with several fusion gene callers. We applied the workflow to the data obtained from ten patients with chronic lymphocytic leukemia (CLL) to describe the consequences of their cSVs in detail. The fusion genes identified by our pipeline were correlated with genomic break-points detected by genomic arrays.

**Results:**

We set up a novel solution for differential gene expression analysis of individual samples and *de novo* fusion gene detection from total RNA-Seq data. The results of the differential gene expression analysis were concordant with results obtained by transcriptomic arrays, which demonstrates the analytical capabilities of our method. We also showed that the consensus fusion gene detection approach was able to identify true positives (TPs) efficiently. Detected coordinates of fusion gene junctions were in concordance with genomic breakpoints assessed using genomic arrays.

**Discussion:**

By****applying our methods to real clinical samples, we proved that our approach for total RNA-Seq data analysis generates results consistent with other genomic analytical techniques. The data obtained by our analyses provided clues for the study of the biological consequences of cSVs with far-reaching implications for clinical outcome and management of cancer patients. The bioinformatic workflow is also widely applicable for addressing other research questions in different contexts, for which transcriptomic data are generated.

## Introduction

Whole-genome sequencing of cancer samples has enabled the identification and detailed description of complex structural variants (cSVs) with chromothripsis being their prime example (Stephens et al., 2011; Rausch et al., 2012). Chromothripsis is characterized by tens to hundreds of clustered genomic rearrangements accompanied by extensive losses of genetic information and arises as a consequence of genomic instability. Approximately 2–3% of tumors bear chromosomes featuring the hallmarks of chromothripsis (Stephens et al., 2011; Kinsella, Patel & Bafna, 2014). Its incidence is variable among tumor types and peaks in brain and bone tumors (Stephens et al., 2011). There is also strong evidence for the presence of chromothripsis and related cSVs in hematological malignancies including chronic lymphocytic leukemia (CLL), the most common leukemia of adults in the Western world.

**Figure 1:**
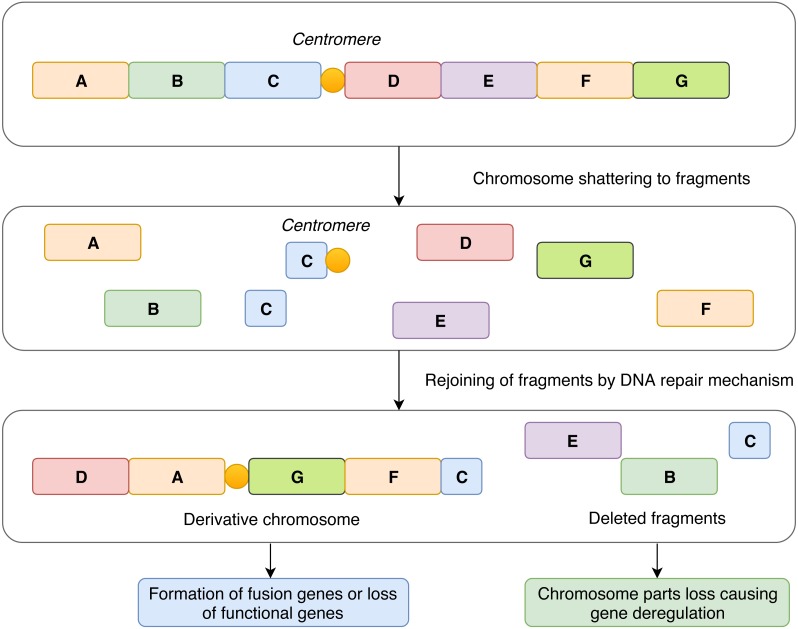
Schematic representation of the mechanism of chromothripsis. During chromothripsis, chromosomes are scattered into hundreds of fragments that persist as intermediates and eventually rejoin together via error-prone DNA repair mechanisms. Such an event can lead to the loss of functional genes and directly or indirectly influence gene expression in affected regions. Another common and frequently observed feature is the formation of aberrant fusion genes, which leads to a change in gene expression and production of fusion proteins with novel structure and functions.

In contrast to the concept of gradual accumulation of chromosomal defects in the cancer genome, it has been assumed that chromothripsis arises in a single catastrophic event. The most widely accepted explanation of chromothripsis origin is based on aberrant mitosis, which is accompanied by physical separation of certain chromosomes in nuclear structures called micronuclei (Zhang et al., 2015; Ly & Cleveland, 2017). Another possible mechanism involved in chromothripsis formation revolves around the generation of so-called breakage-fusion-bridge cycles (Lo et al., 2002) leading to the occurrence of dicentric chromosomes that are disrupted during cell division. This is related to telomere shortening and, consequently, to the absence of telomeres at chromosome ends which enables chromosome fusion (Maciejowski et al., 2015; Ernst et al., 2016). All these events lead to multiple clustered chromosomal aberrations that feature a unique pattern in every affected case and alter the expression of genes in an impaired cell (Fig. 1).

Next-generation sequencing (NGS) serves as a powerful tool for describing any abnormalities occurring in the genome and has also been instrumental in discovering and describing cSVs. However, in many NGS experiments, consequent bioinformatic analysis remains challenging. Publicly available tools are often developed for a specific purpose with limitations in different experimental settings and do not take into account all genomic rearrangements that might be present in a sample. Moreover, when attempting to describe the impact of cSVs on cell phenotype, each case should be considered independently. The reason for such an approach is that, although there are a limited number of processes causing cSVs among individual cases, they affect different genomic loci with different impact. Thus, we developed a statistical approach for differential gene expression analysis from total RNA-Seq data, which is based on the comparison of individual cases within a tested dataset. Applying the method on a set of ten CLL samples, our strategy provided highly convincing gene candidates. In the next step, we focused on the identification of fusion genes and their stemming breakpoints from total RNA-Seq data. Due to the high rate of FP results of different methods, we developed a pipeline combining available analytic tools to maximize TP rate in dataset. Results were then cross-checked with data from genomic arrays in order to assess the sensitivity and specificity of our method.

## Materials & Methods

### Data generation and preprocessing

Our pipelines were developed and tested on total RNA-Seq data generated from ten CLL cases (T1-T10) with cSVs that were previously detected using genomic CytoScan™ HD Arrays and analyzed with Chromosome Analysis Suite (Thermo Fisher Scientific). These cases were identified during the long-term clinical research at the University Hospital Brno and were classified as chromothripsis as they showed clustered copy number alterations on a limited number of chromosomes. Genomic breakpoint localization was extracted for all detected copy number variants and losses of heterozygosity. Total RNA-Seq libraries were prepared with TruSeq^®^ Stranded Total RNA kit (Illumina) with Ribo-Zero ribosomal RNA depletion and sequenced using an Illumina HiSeq 2500 machine producing 125bp long pair-end reads. Sequencing read quality was evaluated in FastQC software. Adapter sequences were trimmed from raw reads using Trimmomatic software (Bolger, Lohse & Usadel, 2014) (version 0.32) according to sequencing facility standards. In parallel, we performed GeneChip^®^ Human Transcriptome Arrays 2.0 (Thermo Fisher Scientific) to complement expression data from the RNA-Seq experiment. These data were then analyzed by the Transcriptome Analysis Console (Thermo Fisher Scientific) by comparing each consecutive sample to all other samples using a one-way ANOVA statistical test to identify a unique expression pattern in each sample. Identified fusion gene junctions and their respective sequencing reads were visualized in the Integrative Genomic Viewer (Thorvaldsdottir, Robinson & Mesirov, 2013).

The study was approved by the Ethical Committee of the University Hospital Brno under the ref. no. 15-31834A. All patients involved in the study provided their written informed consent to the research use of their samples.

### RNA-Seq data processing for differential genes expression analysis

Processed reads were mapped to the hg38 human genome reference using STAR, a splice-aware aligner (Dobin et al., 2013). We chose the genome reference over the transcriptome as recommended by best practices for RNA-Seq data when non-canonical junctions and fusion transcripts are of interest (Conesa et al., 2016). The parameters of the STAR aligner were set to default settings according to best practices.

Gene expression analysis from RNA-Seq data is based on counting reads covering gene regions (defined by reference relative GTF file) and statistical analysis of these read counts. In the first step, we used an htseq-count script (Anders, Pyl & Huber, 2015), which efficiently counts reads that align to or overlap with more than one gene. For this purpose, the software parameter *union* was applied. The reads were then assigned to gene regions irrespective of DNA strand orientation. A read count table was used as an input for consequent statistical analysis of differential gene expression; low-expressed genes were filtered out from the dataset to decrease dataset complexity and avoid FP. Of the ten CLL cases in our cohort, we expected at least six samples to be covered by at least one read. However, these criteria can be modified in the pipeline according to the actual dataset analyzed. Pre-filtered read count table was then normalized using the *rpkm()* function from edgeR Bioconductor package (Robinson, McCarthy & Smyth, 2010). The function applies the RPKM (reads per kilobase per million mapped reads) method, which performs data normalization based on a read length and a total number of sequencing reads with respect to gene length. Obtained log_2_ scale normalized expressions were subjected to further computational steps, for which we developed a novel statistical pairwise comparison (PComp) approach that allows obtaining a gene expression profile of individual samples.

### Design and evaluation of novel statistical approach PComp

**Figure 2:**
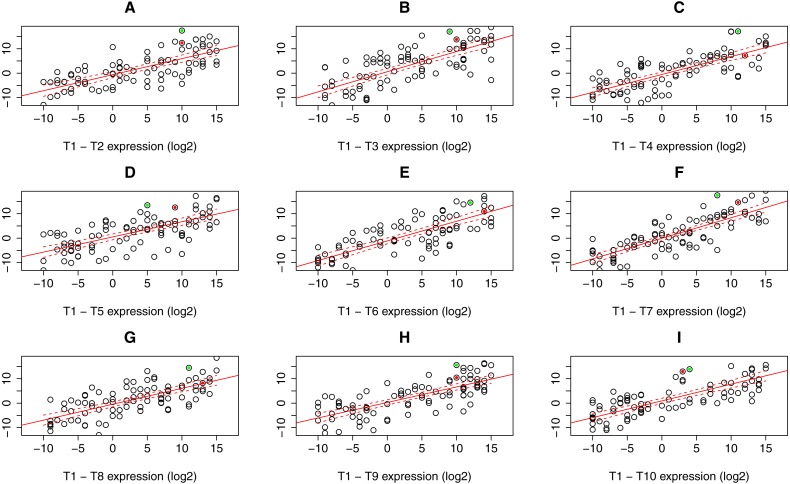
Expression data plots demonstrating our statistical algorithm PComp for differential gene expression assessment. A solid red line represents the linear regression line, while dashed red curves delimit 99.9% confidence bands (A–I). Selected expressed genes identified in an RNA-Seq experiment for sample T1 are highlighted as green and red dots. The green dots represent a gene that is considered significantly differentially expressed in all sample pair combinations, i.e., the gene is outside the confidence band with *p* < 0.001 for all pairs. The gene highlighted as red dots is not significantly differentially expressed when comparing T1 sample to T4, T6, and T8 (C, E, G), thus not differentially expressed as a whole. Down/up-regulation was taken into account.

For the read count (expression) table resulting from RNA-Seq data processing steps, linear regression model with confidence bands containing expression values for all possible sample combinations (i.e., T1–T2, …, T1–T10) was applied to identify gene outliers in particular sample. Only genes constantly lying outside these bands in all possible sample combination were stored. In the next step, one sample *t*-test was applied to assess the *p*.value to every single gene outlier in a given sample according to the gene expression in the rest of the samples. All gene candidates with significant *p*.value that can be specified manually (default 0.05) were considered as differentially expressed. Graphical representation of PComp algorithm is depicted in Fig. 2 with artificial expression values. Obtained results were compared to the transcriptomic array results representing a gold standard for gene expression analysis.

RNA-Seq data were also subjected to a differential gene expression analysis using widely used and well-established algorithm *limma* (Ritchie et al., 2015) to gain an insight on overall performance of our PComp tool. Using *limma* we compared gene expression in a single sample (group 1) with all other samples (group 2) in all possible combinations and correlated the results to the PComp output.

### Identification of gene fusions in cSVs cases from RNA-Seq

Based on a literature search (Liu et al., 2016), four state of the art tools for gene fusion detection—EricScript (Benelli et al., 2012), JAFFA (Davidson, Majewski & Oshlack, 2015), FusionCatcher (Nicorici et al., 2014), and TopHat-fusion (Kim & Salzberg, 2011)—were tested in our approach. TopHat-fusion was excluded due to a high rate of FP results and time-consuming computation in comparison to other methods. Thus, three tools—EricScript, JAFFA, and FusionCatcher—were run in parallel in our bioinformatic workflow for fusion gene identification; default settings were used for all of them. Although read alignment algorithms differ among the selected tools (Kent, 2002; Li & Durbin, 2009; Langmead & Salzberg, 2012; Dobin et al., 2013), the utilized computational algorithms follow similar concept (Kumar et al., 2016). In general, the mapping step consists of two alignments, one to the hg38 reference and the other to putative junction reference for each potential fusion. During these steps, unmapped and discordant pair-end reads that were mapped uniquely to different loci of the genome were identified, and a library of putative fusion junctions was derived. Reads were then realigned to the putative fusion junction sequences and annotated, a split-read signature (reads spanning fusion junctions) was recognized, and potential fusion genes were reported.

**Figure 3:**
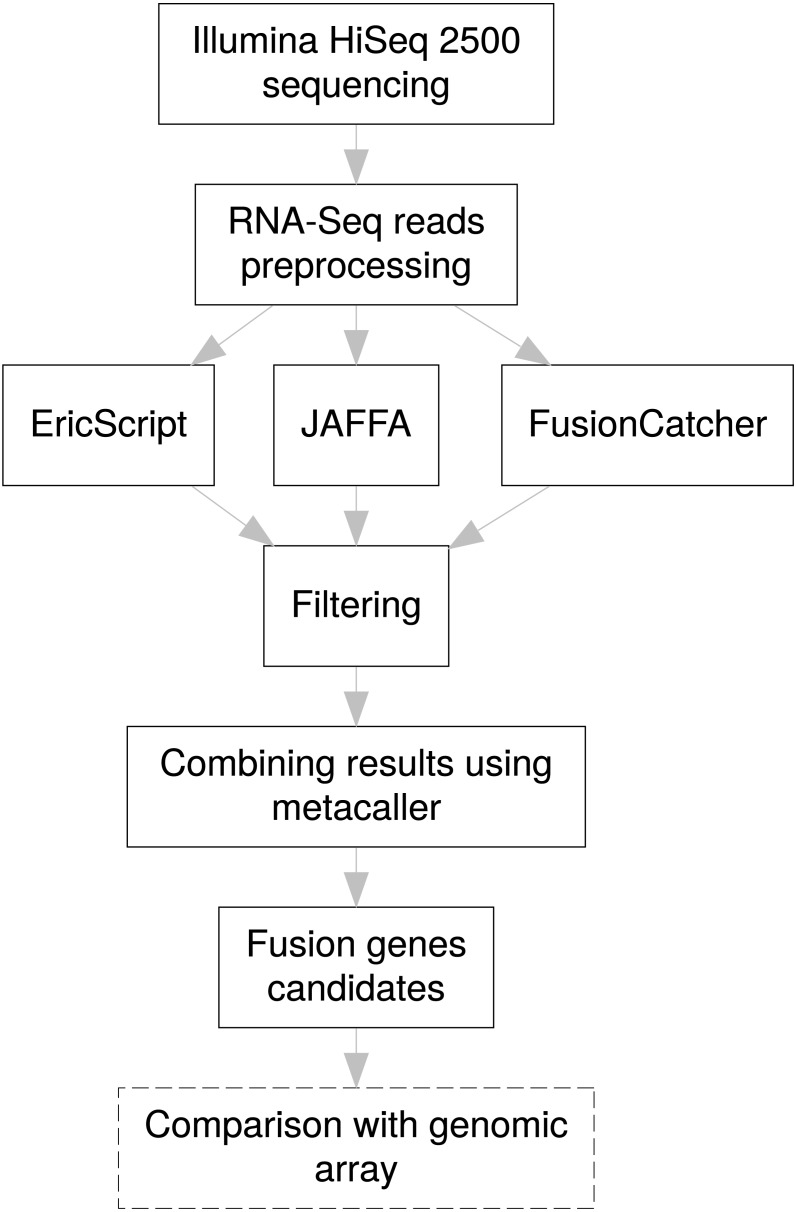
Pipeline design for fusion gene identification. Schematic overview of RNA-Seq fusion gene identification pipeline summarizes all steps of the procedure. Comparison with results of the genomic arrays is an optional manual step, independent on the pipeline, which can serve as a secondary confirmation of the results.

For efficient fusion gene detection, we developed an in-house meta-caller, which combines results from the selected tools into a consensus call. Each piece of software applies various metrics enabling classification of fusions to confidence subsets. In the first meta-caller step, detected fusions were filtered in individual callers as follows: for EricScript fusions with an Eric score (ES) of over 0.90, for JAFFA only fusions with the “HighConfidence” tag, and for FusionCatcher only high confidence fusions. In the consequent consensus call only fusions that passed at least two callers were kept which allowed increasing TP fusion rate and removal of FP fusions arising as specific artifacts of an individual caller algorithm (i.e., read alignments errors). The whole fusion gene detection pipeline is schematically visualized in the Fig. 3.

### Validation of gene fusions by overlap with genomic arrays

Finally, to estimate TP and FP rates of the individual callers and the meta-caller, coordinates of fusions detected in RNA-Seq data were compared with the coordinates of genomic breakpoints detected by the genomic arrays. The interval of ± 100 kb around the breakpoints was applied in order to adjust to the array resolution and to the fact that the breakpoints can be located in introns, whereas fusions in RNA-Seq data appear in exon-exon boundaries. All potentially TP fusions identified by this approach were inspected visually in the array results.

### Implementation of analytical algorithms

Algorithms and procedures of our novel solution were implemented in the R programming language. The meta-caller and the tool for differential expression are both freely available at GitHub (https://github.com/Hynst/WTS_cSVs_analysis), where the source codes are accessible for download. No installation is needed, as all dependencies (i.e., R packages and libraries) are installed automatically to the R environment.

## Results

### PComp performance in differential gene expression analysis

We used PComp approach to identify differentially expressed genes on the level of individual samples. We followed a linear regression model and estimated a linear regression line with 99.9% confidence bands (1 − *p* = 0.999, i.e.,  *p* = 0.001) for each pair of samples. All genes lying outside the confidence bands were suggested as potentially differentially expressed candidates. We repeated this procedure for each sample pair combination and obtained nine sets of potential candidates per sample. Genes occurring in all nine sets were considered as differentially expressed for a given sample. By applying one sample *t*-test we assessed the differential expression of a given gene in a given sample according to the rest of the samples. We considered all gene candidates in particular sample with a *p* < 0.001 by one sample *t*-test to be differentially expressed. Using this approach, we found 15,200 differentially expressed genes in total (Table 1).

**Table 1:** Differentially expressed genes identified by PComp in RNA-Seq data and transcriptomic array analyses. For RNA-Seq data pairwise comparison (PComp) analysis was applied to identify deregulated genes. Numbers in parentheses represent deregulated genes including also unannotated transcripts.

**Sample**	**RNA-Seq Expression Analysis (PComp)**			**GeneChip Human Transcriptome Array 2.0**			**Method overlap**		
	up	down	total	up	down	total	up	down	total
T1	1,534	552	2,086	707 (956)	399 (909)	1,106	351	29	380
T2	539	66	605	253 (660)	83 (191)	336	54	1	55
T3	314	82	396	124 (293)	92 (188)	216	22	3	25
T4	1,768	657	2,425	482 (882)	154 (197)	636	207	66	273
T5	1,572	355	1,927	567 (1,268)	77 (127)	644	78	21	99
T6	1,346	258	1,604	644 (1,405)	134 (178)	778	178	38	216
T7	1,865	245	2,110	423 (804)	186 (439)	609	164	15	179
T8	1,855	419	2,274	1,446 (2,173)	428 (821)	1,874	682	55	737
T9	544	96	640	124 (384)	92 (110)	216	27	3	30
T10	968	165	1,133	213 (418)	50 (89)	263	45	5	50
TOTAL	12,305	2,895	15,200	4,983	1,695	6,678	1,808	236	2,044

We inspected the results from RNA-Seq obtained using the PComp method and compared them to the results of transcriptomic arrays analyzed by one-way ANOVA. In the ten CLL samples tested, we identified 12,492 deregulated transcripts (including also unannotated ones) using transcriptomics arrays and extracted only annotated gene transcripts which resulted in 6,678 deregulated genes. We created an overlap between PComp and transcriptomic array results and found 1,808 significantly upregulated and 236 significantly downregulated gene candidates (Table 1). We also studied whether the results were concordant between the methods in terms of assigning genes as up-/downregulated and observed good concordance of the methods ranging 97.44–100% for individual samples (Table S1).

**Figure 4:**
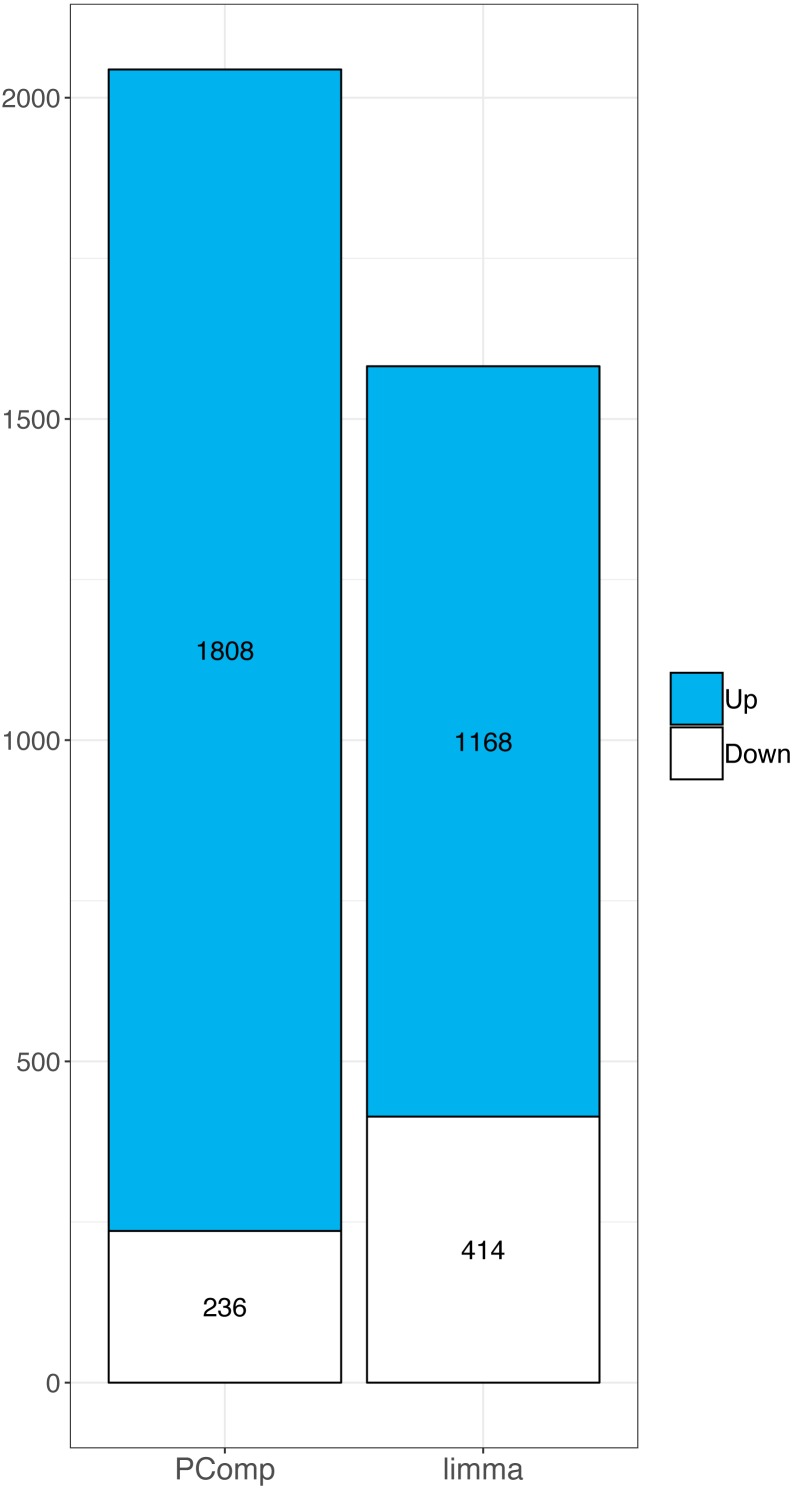
Comparison of PComp vs. *limma* statistical analysis of RNA-Seq data overlapped with results of the transcriptomic arrays. Graphical representation of upregulated (blue) and downregulated (white) genes as identified by PComp and *limma* after overlapping the data with transcriptomic array outputs. PComp was capable of identifying higher number of differentially expressed genes concordant with transcriptomic arrays.

Similarly, in RNA-Seq data we identified 14,803 deregulated genes using *limma*. When we overlapped array data with *limma* results we found 1168 upregulated and 414 downregulated genes (Table S2). We compared the overlap rate of transcriptomic array with PComp and *limma* data, respectively, in terms of up-/downregulated genes. Both methods performed differently, we observed 14.7% and 28.9% upregulated genes, and 8.2% and 4.1% downregulated genes using PComp and limma, respectively. Finally, we observed larger overlap between transcriptomic array and PComp approach (Fig. 4), where 2044 deregulated genes were identified in total (compared to 1,585 identified by *limma*).

### Meta-caller approach efficiency of fusion gene identification

In RNA-Seq data, we pre-filtered results of the individual pieces of software in the first meta-caller step to create a fusion gene subset with high confidence. No explicit pre-filtering of lowly expressed fusions was done in order to allow for the capture of lowly expressed fusion genes (i.e., the number of reads spanning fusion junction ≥1). In the next step of the meta-caller, results obtained by ≥2 callers were combined into a consensus call. Following these steps, our pipeline was capable of identifying 40 fusion genes which were potentially TP with high confidence across the ten samples (Table S3). Further, we used breakpoint coordinates generated using genomic arrays and compared them with those identified by RNA-Seq to test TP rates of our pipeline. Only fusions where at least one fusion gene partner was located in a genomic breakpoint were considered as potentially TP. Genomic array data corresponded to RNA-Seq breakpoints in 19 of the 40 cases. These uniquely expressed TP fusions were identified in eight of the ten CLL patients with 1 to 7 fusions per patient.

Considering candidates that were not supported by genomic arrays (21 of 40 features), potential fusions ZMYM5-PSPC1, HACL1-COLQ, MFSD7-ATP5I, and CYTIP-ERMN, occurring recurrently in six, three, three and two instances, respectively, were considered FP because they represented neighboring genes (read-through). Similarly, YWHAZ-ZNF706, LINCPINT-MKLN1, and NFATC3-PLA2G15 were also considered FP due to their adjacent localization in the genome, although not observed recurrently. Altogether, 17 of 21 fusions were considered FP. The remaining four fusions were not confirmed by the independent method; in all of them, at least one fusion partner appeared recurrently in the data, however, with a different partner. All of the four fusions could still represent TP resulting from balanced translocations or inversions that were not detectable by genomic arrays.

To assess the ability of the meta-caller to increase TP rate, we inspected overlap between individual callers and genomic arrays (Table 2). The meta-caller was superior to a single caller approach with the overall 47.5% TP rate, while JAFFA, FusionCatcher, and EricScript showed 9.4%, 8.2%, and 0.5% TP rate, respectively. Still individual callers identified TP fusions (as designated based on the overlap with genomic array results) which were missed by the meta-caller because they were supported only by one caller. Altogether 29 TP fusions were detected by all callers and meta-caller with the highest number of 22 TP fusions by FusionCatcher (Table S4). FusionCatcher and the meta-caller did not show any FP when overlapped with genomic arrays. Detailed results of this comparison are depicted in Table 3. Taking overall TP rates of the callers into account, the meta-caller appears as an efficient tool for fusion gene detection.

**Table 2:** True positive rate of fusion genes identified using the individual pieces of software and the meta-caller.

**Sample**	**EricScript**			**JAFFA**			**FusionCatcher**			**meta-caller**		
	fusions overall	TP	TP rate (%)	fusions overall	TP	TP rate (%)	fusions overall	TP	TP rate (%)	fusions overall	TP	TP rate (%)
T1	207	3	1.45	37	5	13.51	48	4	8.33	9	3	33.33
T2	275	0	0	22	1	4.55	19	2	10.53	2	1	50.00
T3	180	0	0	13	0	0	18	0	0	1	0	0
T4	188	1	0.53	20	0	0	7	1	14.29	2	1	50.00
T5	211	2	0.95	8	2	25.00	10	1	10.00	3	2	66.67
T6	165	0	0	16	1	6.25	14	1	7.14	1	1	100.00
T7	182	0	0	15	3	20.00	31	4	12.90	6	3	50.00
T8	133	0	0	12	0	0	39	1	2.56	1	0	0
T9	336	0	0	30	1	3.33	29	1	3.45	4	1	25.00
T10	189	4	2.12	29	6	20.69	53	7	13.21	11	7	63.64
**TOTAL**	**2,066**	**10**	**0.48**	**202**	**19**	**9.41**	**268**	**22**	**8.21**	**40**	**19**	**47.50**

**Table 3:** Overlap between gene fusions identified by RNA-Seq and genomic breakpoints detected by genomic arrays. Numbers of true positive (TP), false positive (FP) and false negative (FN) results evaluated using the individual callers and the meta-caller in a set of total 29 fusion genes overlapping with genomic breakpoints.

**Software**	**Fusions identified by overlap between RNA-Seq and arrays**	**TP**	**FP**	**FN**
EricScript	17	10	7	19
FusionCatcher	22	22	0	7
JAFFA	21	19	2	10
meta-caller	19	19	0	10

## Discussion

Several mechanisms of the influence of cSVs on cancer development have been suggested. NGS techniques provide an opportunity to unravel them by focusing on changes occurring at both the DNA and the RNA level. However, there still is a need for designing efficient pipelines and algorithms for consequent bioinformatic analyses. In this article, we introduce our approach for RNA-Seq data analysis in cases with cSVs. We tested the proposed workflow on a set of ten CLL patients with chromothripsis to validate the efficiency of our method.

Although cSVs reminiscent of chromothripsis likely share a common mechanism of origin across cSV cases, they appear in various genomic loci and, consequently, they lead to diverse expression profiles which makes it very difficult to select an appropriate biological group as a control. Unfortunately, the majority of conventional tools for differential gene expression (Robinson, McCarthy & Smyth, 2010; Love, Huber & Anders, 2014) are designed to distinguish two (or more) biological groups (i.e., treated vs. untreated), with each of them bearing uniform expression profiles. To the best of our knowledge there is no well-established tool for individual sample expression analysis. Thus, we developed PComp, a statistical framework for RNA-Seq differential gene expression analysis, that allows us to describe the impact of genomic changes through identification of exclusive features related to an actual cSV pattern in a given case. We believe this is the first approach developed and fully dedicated to address this challenge. A set of significantly deregulated genes, according to the regression model and one sample *t*-test, is the output of the analysis. PComp provides solid results comparable with transcriptomic array analysis and in our comparison performed better than *limma* tool, which is used widely for RNA-Seq data analysis. Although in our experimental setup we expected unique expression profiles for individual samples of our sample set, it is possible to specify the number of samples that can share expression similarities within the tool. We believe that the PComp approach is versatile and applicable to various RNA-Seq experimental designs and biological questions where a single sample expression profile is desired. Last but not least, the PComp tool is freely available on GitHub and easy to use by the potential users on their own data.

Another common feature of cSV case is the formation of fusion genes originating in chromosome shattering and consequent segment rejoining. Approximately 20% of fusion genes are expressed (Boeva et al., 2013), which in theory leads to the assembly of *de novo* proteins with aberrant structure and function. Aberrant protein products of fusion genes can influence important biological processes through direct or indirect regulation of expression of various genes which can eventually be reflected in the overall expression profile. Effective screening for such events in various diagnoses could help with identifying specific, potentially targetable disease markers (Holderfield et al., 2014).

Current approaches for fusion gene identification have many limitations. Among them, a high FP rate represents a significant obstacle, often hindering proper analysis (Boeva et al., 2013). Apart from that, in cSV cases, one must also deal with fundamental structural complexity. Thus, a need for the development of new software and tools to address these challenges is obvious. In our test cohort, we tested four state-of-the-art tools for fusion gene identification; from which we selected EricScript, JAFFA, and FusionCatcher based on their performance. To increase the precision of fusion gene identification and to overcome possible technical issues potentially resulting in FPs, we applied a method of consensus fusion gene calling where we combined results filtered to confidence subsets from the selected methods (Liu et al., 2016) in our in-house meta-caller. Consensus calling improves the precision of results, increases overall TP rate and significantly saves time, which needs to be dedicated to manual inspection of the results. In the consensus calling approach, a low rate of FP results is the biggest advantage, on the other hand, some TPs can be lost due to their detection by a single caller. Notwithstanding, there has not been a tool providing an optimal solution for handling potential TPs and FPs and considering the overall TP rates of the tested callers, our meta-caller appears as a good solution for fusion gene detection.

Our analytical procedure was able to detect 40 fusion genes from RNA-Seq data of the ten CLL cases. We localized genomic breakpoints, compared the data with coordinates assessed by genomic arrays and found high overlap between the methods. We have also found cases where no TP fusions were identified; we hypothesize that in these cases fusion genes were either not present, transcribed or were below the detection limit of RNA-Seq experiment. We identified several FPs in our RNA-Seq results. The most common reason for their detection was adjacent localization of the partners in the genome leading to their coupled transcription. Some of them occurred in the dataset recurrently, which is highly unlikely in cases with cSVs differing considerably among the samples. However, we have also noted genes recurring in fusions but with different partners localized even on different chromosomes. We did not consider these directly as FPs though they were not confirmed by other methods, since genomic arrays may produce false negative results due to balanced translocations or inversions.

## Conclusions

Complex structural variants, such as chromothripsis, have a significant impact on cellular physiology and thus also dramatically influence the biological features of a cell. Advanced experimental approaches, e.g., total RNA-Seq, has enabled the study of the causes and consequences of chromosomal shattering in detail, however, the bioinformatic component of the analysis still needs improvement. We developed bioinformatic pipelines for differential gene expression analysis and fusion gene identification in RNA-Seq data. We applied the pipelines to the set of CLL cases with chromothripsis and obtained results highly consistent with other experimental approaches (transcriptomic and genomic arrays). In our test dataset, the PComp tool outperformed well-established *limma* approach and the meta-caller dramatically increased overall TP rate of fusion gene detection and allowed for effective FP filtering. The general algorithm and the steps of our pipelines are broadly applicable in many experimental setups.

##  Supplemental Information

10.7717/peerj.7071/supp-1 Table S1 Correlation of up-/downregulated genes identified by PComp analysis and transcriptomic arrays The table depicts comparison of numbers of genes identified as differentially expressed using PComp approach for RNA-Seq data analysis and one-way ANOVA statistical test for transcriptomic array. The concordance was calculated as percentage of genes assigned concordantly between the methods.

10.7717/peerj.7071/supp-2 Table S2 Differentially expressed genes identified by *limma* in RNA-Seq data and transcriptomic array analyses For RNA-Seq data, *limma* analysis was applied to identify deregulated genes. Numbers in parentheses represent deregulated genes including also unannotated transcripts.

10.7717/peerj.7071/supp-3 Table S3 List of potential gene fusions identified by the meta-caller Overall 40 fusion genes in 8 out of 10 tested samples were identified using consensus meta-caller step. True/false positive gene fusions were assessed manually based on visual inspection of genomic array data (NA, not assessed; FP, false positive; TP, true positive).

10.7717/peerj.7071/supp-4 Table S4 Full list of TP fusion genes identified in all callers Overview of 29 TP fusion genes identified by all used callers, i.e., EricScript (eric), FusionCatcher (FC) and JAFFA. Fusion genes identified by meta-caller had to be present in the results of ≥2 callers.

10.7717/peerj.7071/supp-5 Supplemental Information 1 MIAME checklist The file contains the description of procedure used for RNA expression analysis using transcriptomic arrays.

10.7717/peerj.7071/supp-6 Supplemental Information 2 MIAME checklist The file contains the description of procedure used for cytogenomic analysis using cytogenetic arrays.

## Additional Information and Declarations

### DNA Deposition

The following information was supplied regarding the deposition of DNA sequences: The data are available at the GEO database: GSE128668.

### Microarray Data Deposition

The following information was supplied regarding the deposition of microarray data: The respective data were uploaded to the GEO database under accession numbers GSE124329 and GSE124330.

### Data Availability

The following information was supplied regarding data availability: Data is available at GitHub:
https://github.com/Hynst/WTS_cSVs_analysis.

## References

[ref-1] Anders S, Pyl PT, Huber W (2015). HTSeq—a Python framework to work with high-throughput sequencing data. Bioinformatics.

[ref-2] Benelli M, Pescucci C, Marseglia G, Severgnini M, Torricelli F, Magi A (2012). Discovering chimeric transcripts in paired-end RNA-seq data by using EricScript. Bioinformatics.

[ref-3] Boeva V, Jouannet S, Daveau R, Combaret V, Pierre-Eugène C, Cazes A, Louis-Brennetot C, Schleiermacher G, Ferrand S, Pierron G, Lermine A, Frio TR, Raynal V, Vassal G, Barillot E, Delattre O, Janoueix-Lerosey I (2013). Breakpoint features of genomic rearrangements in neuroblastoma with unbalanced translocations and chromothripsis. PLOS ONE.

[ref-4] Bolger AM, Lohse M, Usadel B (2014). Trimmomatic: a flexible trimmer for Illumina sequence data. Bioinformatics.

[ref-5] Conesa A, Madrigal P, Tarazona S, Gomez-Cabrero D, Cervera A, McPherson A, Szcześniak MW, Gaffney DJ, Elo LL, Zhang X, Mortazavi A (2016). A survey of best practices for RNA-seq data analysis. Genome Biology.

[ref-6] Davidson NM, Majewski IJ, Oshlack A (2015). JAFFA: high sensitivity transcriptome-focused fusion gene detection. Genome Medicine.

[ref-7] Dobin A, Davis CA, Schlesinger F, Drenkow J, Zaleski C, Jha S, Batut P, Chaisson M, Gingeras TR (2013). STAR: ultrafast universal RNA-seq aligner. Bioinformatics.

[ref-8] Ernst A, Jones DTW, Maass KK, Rode A, Deeg KI, Jebaraj BMC, Korshunov A, Hovestadt V, Tainsky MA, Pajtler KW, Bender S, Brabetz S, Gröbner S, Kool M, Devens F, Edelmann J, Zhang C, Castelo-Branco P, Tabori U, Malkin D, Rippe K, Stilgenbauer S, Pfister SM, Zapatka M, Lichter P (2016). Telomere dysfunction and chromothripsis. International Journal of Cancer.

[ref-9] Holderfield M, Deuker MM, McCormick F, McMahon M (2014). Targeting RAF kinases for cancer therapy: BRAF-mutated melanoma and beyond. Nature Reviews. Cancer.

[ref-10] Kent WJ (2002). BLAT—the BLAST-like alignment tool. Genome Research.

[ref-11] Kim D, Salzberg SL (2011). TopHat-Fusion: an algorithm for discovery of novel fusion transcripts. Genome Biology.

[ref-12] Kinsella M, Patel A, Bafna V (2014). The elusive evidence for chromothripsis. Nucleic Acids Research.

[ref-13] Kumar S, Vo AD, Qin F, Li H (2016). Comparative assessment of methods for the fusion transcripts detection from RNA-Seq data. Scientific Reports.

[ref-14] Lo AW l, Sabatier L, Fouladi B, Pottier G, Ricoul M, Mumane JP (2002). DNA amplification by breakage/fusion/bridge cycles initiated by spontaneous telomere loss in a human cancer cell line. Neoplasia.

[ref-15] Langmead B, Salzberg SL (2012). Fast gapped-read alignment with Bowtie 2. Nature Methods.

[ref-16] Li H, Durbin R (2009). Fast and accurate short read alignment with Burrows-Wheeler transform. Bioinformatics.

[ref-17] Liu S, Tsai W-H, Ding Y, Chen R, Fang Z, Huo Z, Kim S, Ma T, Chang T-Y, Priedigkeit NM, Lee AV, Luo J, Wang H-W, Chung I-F, Tseng GC (2016). Comprehensive evaluation of fusion transcript detection algorithms and a meta-caller to combine top performing methods in paired-end RNA-seq data. Nucleic Acids Research.

[ref-18] Love MI, Huber W, Anders S (2014). Moderated estimation of fold change and dispersion for RNA-seq data with DESeq2. Genome Biology.

[ref-19] Ly P, Cleveland DW (2017). Rebuilding Chromosomes After Catastrophe: emerging Mechanisms of Chromothripsis. Trends in Cell Biology.

[ref-20] Maciejowski J, Li Y, Bosco N, Campbell PJ, De Lange T (2015). Chromothripsis and kataegis induced by telomere crisis. Cell.

[ref-21] Nicorici D, Satalan M, Edgren H, Kangaspeska S, Murumagi A, Kallioniemi O, Virtanen S, Kilkku O (2014). FusionCatcher—a tool for finding somatic fusion genes in paired-end RNA-sequencing data. bioRxiv.

[ref-22] Rausch T, Jones DTW, Zapatka M, Stütz AM, Zichner T, Weischenfeldt J, Jäger N, Remke M, Shih D, Northcott PA, Pfaff E, Tica J, Wang Q, Massimi L, Witt H, Bender S, Pleier S, Cin H, Hawkins C, Beck C, Von Deimling A, Hans V, Brors B, Eils R, Scheurlen W, Blake J, Benes V, Kulozik AE, Witt O, Martin D, Zhang C, Porat R, Merino DM, Wasserman J, Jabado N, Fontebasso A, Bullinger L, Rücker FG, Döhner K, Döhner H, Koster J, Molenaar JJ, Versteeg R, Kool M, Tabori U, Malkin D, Korshunov A, Taylor MD, Lichter P, Pfister SM, Korbel JO (2012). Genome sequencing of pediatric medulloblastoma links catastrophic DNA rearrangements with TP53 mutations. Cell.

[ref-23] Ritchie ME, Phipson B, Wu D, Hu Y, Law CW, Shi W, Smyth GK (2015). limma powers differential expression analyses for RNA-sequencing and microarray studies. Nucleic Acids Research.

[ref-24] Robinson MD, McCarthy DJ, Smyth GK (2010). edgeR: a Bioconductor package for differential expression analysis of digital gene expression data. Bioinformatics.

[ref-25] Stephens PJ, Greenman CD, Fu B, Yang F, Bignell GR, Mudie LJ, Pleasance ED, Lau KW, Beare D, Stebbings LA, McLaren S, Lin M-L, McBride DJ, Varela I, Nik-Zainal S, Leroy C, Jia M, Menzies A, Butler AP, Teague JW, Quail MA, Burton J, Swerdlow H, Carter NP, Morsberger LA, Iacobuzio-Donahue C, Follows GA, Green AR, Flanagan AM, Stratton MR, Futreal PA, Campbell PJ (2011). Massive genomic rearrangement acquired in a single catastrophic event during cancer development. Cell.

[ref-26] Thorvaldsdottir H, Robinson JT, Mesirov JP (2013). Integrative Genomics Viewer (IGV): high-performance genomics data visualization and exploration. Briefings in Bioinformatics.

[ref-27] Zhang C-Z, Spektor A, Cornils H, Francis JM, Jackson EK, Liu S, Meyerson M, Pellman D (2015). Chromothripsis from DNA damage in micronuclei. Nature.

