# Supplementary material for: Bioinformatic pipelines for whole transcriptome sequencing data exploitation in leukemia patients with complex structural variants
Source: PeerJ. 2019 Jun 12;7:e7071. doi: 10.7717/peerj.7071 (PMC6571010; doi:10.7717/peerj.7071)
Supplement: Supplemental Information 1 — The file contains the description of procedure used for RNA expression analysis using transcriptomic arrays. [file peerj-07-7071-s005.docx]

**Miame Checklist**

(Sections in **BOLD** represent what each researcher must record about

every slide and/or experiment)

Part 1 Experiment description

*“The minimal information required in this section includes the type of the experiment (such as normal-versus-diseased comparison, time course, dose response, and so on) and the experimental variables, including parameters or conditions tested (such as time, dose, genetic variation or response to a treatment or compound).”*

*“this section specifies the experimental relationships between the array and sample entities—that is, which samples and which arrays were used in each hybridization assay. Each of these will be assigned unique identifiers that are cross-referenced with the information provided in the following sections.”*

-         **mouse type**

-         **experimental variables (runners vs. non-runners, high fat vs. low fat)**

-         **n-count**

-         **tissues used for slide**

-         **mouse age, and other variables (wean weight, pooled samples, etc.)**

10 human samples (T1-T10; 8 males, 2 females) from patients with chronic lymphocytic leukemia bearing extensive genomic rearrangements

Tissue used – peripheral blood

Part 2 Array design.

*“The aim of this section is to provide a systematic definition of all arrays used in the experiment, including the genes represented and their physical layout on the array.”*

*“The array-type definition includes information common to all arrays of a particular type (such as glass-slide spotted with PCR-amplified cDNA clones) as well as precise descriptions of the physical content of each element (spot or feature). This section consists of three parts: (i) a description of the array as a whole (such as platform type, provider and surface type); (ii) a description of each type of element or spot used (properties that are typically common to many elements, such as 'synthesized oligo-nucleotides' or 'PCR products from cDNA clones'); and (iii) a description of the specific properties of each element, such as the DNA sequence and, possibly, quality-control indicators.”*

-         **Array series**

-         **Deconvoluted spot list with gene names**

-         **Array type (mouse, human, cDNA, oligo, number of genes)**

-         **Array size**

-         **Slide type (and coating)**

GeneChip® Human Transcriptome Array 2.0, Affymetrix

Part 3 Samples

*“The MIAME 'sample' concept represents the biological material (or biomaterial) for which the gene expression profile is being established. This section is divided into three parts which describe the source of the original sample (such as organism taxonomy and cell type) and any biological*in vivo*or*in vitro*treatments applied, the technical extraction of the nucleic acids, and their subsequent labeling.”*

-         **Cy3/Cy5 labels for tissues**

-         **Dye swap? Or reference control?**

-         **Labelling protocol used**

-         **Sample extraction protocol used**

-         **Amount of sample labelled**

The arrays were processed according to the manufacturer’s recommendation.

Sample extraction was performed using TriReagent (MRC) with isopropanol precipitation. Input amount was 500 pg of RNA.

Part 4 Hybridizations

*“This section defines the laboratory conditions under which the hybridizations were carried out. Other than a free-text description of the hybridization protocol, MIAME requires that a number of critical hybridization parameters are explicitly specified: choice of hybridization solution (such as salt and detergent concentrations), nature of the blocking agent, wash procedure, quantity of labeled target used, hybridization time, volume, temperature and descriptions of the hybridization instruments.”*

-         **Hybridization protocol**

-         **ALL modifications and deviations from the protocol**

-         **Manual hybridization or automatic chamber?**

-         **Number of slides done at the same time**

-         **Hyb time**

-         **Number of washes**

-         **Amount of labelled sample hybridized**

-         **Labelling efficiency**

Hybridization followed the manufacturer’s recommendation; no modifications were done.

10 arrays processed at the same time, washes performed in GeneChip Fluidic Station 450.

Part 5 Measurements

*“Image data should be provided as raw scanner image files (such as TIFF), accompanied by scanning information that includes relevant scan parameters and laboratory protocols.”*

-         **Which version of scanner software used**

-         **Laser power for scan**

-         **Instrument model numbers**

-         **Must save original .tiff format images (composite image is optional)**

GeneChipTM Scanner 3000 7G was used for scanning.

Raw data were uploaded to the GEO database.

*For each experimental image, a microarray quantification matrix contains the complete image analysis output as directly generated by the image analysis software (normally provided as separate spreadsheet-type files). Note that for a given image this is a 2D matrix, where array elements (spots or features) constitute one dimension and quantification types (such as mean and median intensity, mean or median background intensity) are the second dimension.*

-         **Normalization protocol**

-         **Does the scanner software subtract background? How much?**

-         **Spot raw values, background intensity, ch1 and 2 intensity, etc.**

-         **Corresponding gene name**

-         **Methods of analysis (MAN, Spotfire, Genespring) be detailed.**

-         **Normalized to controls? Controls removed? All normalization parameters**

-         **Name of Images, Experiment, and location of files.**

-         **Lowess or other normalization if used (and parameters)**

All controls recommended by the manufacturer in the protocol were used. Data were processed using Expression Console™ Software and Transcriptome Analysis Console as recommended by the manufacturer. Processed data were uploaded to the GEO database.

*Finally, the gene expression matrix (summarized information) consists of sets of gene expression levels for each sample. If microarray quantification matrices can be considered spot/image centric, then the gene expression matrix is gene/sample centric. At this point, the expression values may have been normalized, consolidated and transformed in any number of ways by the submitter in order to present the data in a form amenable to scientific analysis. Rather than attempting to impose a standard for gene expression values, MIAME indicates preferred detailed specifications of all numerical calculations applied to unprocessed quantifications in (b) that have led to the data in (c). Experimenters are encouraged, though not required, to provide reliability indicators (such as s.d.) for each data point.*

-         **Output file**

-         **Normalized ratios**

-         **Numerical manipulations**

-         **Cut off values**

 Data were processed as recommended by the manufacturer. All further manipulations were described in detail in the methods section of the manuscript.

Part 6 Normalization controls

*“A typical microarray experiment involves a number of hybridization assays in which the data from multiple samples are analyzed to identify relative changes in expression levels, identify differentially expressed genes and, in many cases, discover classes of genes or samples having similar patterns of expression.”*

-         **Hypothesis**

-         **Gene expression patterns found**

-         **Controls used, normalization methods used (see above)**

All controls recommended by the manufacturer in the protocol were used.
